# Coexistence of papillary thyroid cancer with Hashimoto thyroiditis

**DOI:** 10.1007/s00423-012-1021-x

**Published:** 2012-10-26

**Authors:** Aleksander Konturek, Marcin Barczyński, Wojciech Wierzchowski, Małgorzata Stopa, Wojciech Nowak

**Affiliations:** 1 Department of Endocrine Surgery, 3rd Chair of General Surgery, Jagiellonian University Medical College, 37 Prądnicka Street, 31-202 Krakow, Poland; 2 Department of Pathology, Jagiellonian University Medical College, 16 Grzegorzecka Street, 31-531 Krakow, Poland

**Keywords:** Hashimoto thyroiditis, Papillary thyroid carcinoma, Benign thyroid disease

## Abstract

**Aims:**

Conflicting data have been reported with regard to Hashimoto thyroiditis (HT) and risk of malignancy. The aim of this study was to evaluate coexistence of papillary thyroid cancer (PTC) with HT.

**Patients and methods:**

This is a retrospective cohort study in which HT was diagnosed in 452 (F/M ratio = 405:47, median age 53.5 ± 12.1 years) of 7,545 patients qualified for thyroidectomy throughout the years 2002 to 2010. Pathological reports were reviewed to identify prevalence of PTC in HT vs. non-HT patients.

**Results:**

PTC was diagnosed in 106 of 452 (23.5 %) HT patients vs. 530 of 7,093 (7.5 %) non-HT patients (*p* < 0.001). Metastases to level VI lymph nodes were observed in 81 of 106 (76.4 %) patients with PTC in HT vs. 121 of 530 (22.8 %) patients with PTC in non-HT disease (*p* < 0.001).

**Conclusions:**

HT was associated with a threefold increase of PTC prevalence as compared to other non-HT thyroid diseases, and the spread of PTC to level VI lymph nodes was four times more frequent in HT than in non-HT patients.

## Introduction

Autoimmune thyroid disease known as Hashimoto thyroiditis (HT) is one of special forms of chronic thyroiditis and the most common non-iatrogenic cause of hypothyroidism. The condition is definitely more prevalent in female population (M/F ratio of 1:10, 1:20), is seen in each age group, and may also affect children and adolescents. Its unclear etiopathogenesis strongly indicates an autoimmune background, associated with T-helper lymphocyte (CD4+) activation by class II human leukocyte antigen system cells (MHC class II: HLA-DR3, HLA-DR4, HLA-DR5). On the one hand, the cells recruit cytotoxic lymphocytes (T_c_, CD8+), thus facilitating a release of cytokines that damage thyroid follicular cells and, on the other hand, activating B lymphocytes; they facilitate production of specific anti-microsomal, anti-thyroglobulin, or anti-TSH receptor antibodies. Described for the first time in 1912, the disease may have two distinct histological forms: atrophic and nodular. Clinically, in the majority of cases, it is characterized by hypothyroidism, although in a small number of patients, it may be preceded by symptoms of hyperthyroidism. The association between coexistence of Hashimoto thyroiditis and papillary thyroid cancer (PTC) was first described by Dailey et al. in 1955. Since that time, attempts have been made at elucidation of this phenomenon. The objective of the present paper was to demonstrate coexistence of HT with an autoimmune background with highly differentiated thyroid cancer and to attempt to determine assumptions for further therapeutic management and prognosis in patients operated on for Hashimoto thyroiditis and papillary thyroid cancer.

An early detection of lesions, a careful selection of a surgical strategy in a referral center, target-based adjuvant therapy, and treatment monitoring may have a significant impact on improvement of therapeutic outcomes and quality of life in patients with the disease [1, 2].

## Materials and methods

A retrospective analysis included 452 patients diagnosed with Hashimoto thyroiditis and coexisting papillary thyroid cancer selected from among 7,545 patients treated surgically in a single clinical referral center in the years 2002–2010. The mean age of the investigated group was 53.5 ± 12.1 years and the F/M ratio was 405:47. The demographics of the investigated group are presented in Table 1. Assessment of the clinical stage was based on the seventh edition of the TNM system (2010), while histopathology results were classified based on a reanalysis of pathology reports pertaining to materials stored in the Histology Department Records. The diagnosis of Hashimoto thyroiditis was defined as the presence of lymphocytic infiltrations in the thyroid parenchyma and stroma, with formation of reaction centers and lymphoid nodules and presence of oxyphilic cells. The lymph nodes were characterized by nonspecific reactive lesions. High levels of anti-peroxidase antibodies confirmed the diagnosis. The extent of primary surgery performed in the presented group included total thyroidectomy with central compartment lymph node dissection in 91 % patients; in the remaining cases, bilateral subtotal thyroid lobectomies were done. Histopathology confirming cancer presence constituted an indication for total resection of the residual thyroid tissue.

**Table 1:** Demographic characteristics of 7,545 patients analyzed in this study

	HT (*n* = 452)	Non-HT (*n* = 7,093)	*p* value
Gender ratio (M/F)	47:405	667:6,426	0.484
Average age (years)	53.5 ± 12.1	52.3 ± 15.5	0.739
Preoperative thyroid volume in ml (by ultrasound), mean ± SD	22.1 ± 18.2	96.2 ± 40.4	<0.001^a^
Preoperative diagnosis, *n* (%)			
Benign thyroid disease (BTD)	421 (93.1)	6,881 (97.0)	<0.001
Positive FNAB for PTC	31 (29.2)	212 (40.0)	<0.001

*BTD* multinodular nontoxic goiter, multinodular toxic goiter, Graves disease, and Hashimoto disease, *FNAB* fine-needle aspiration biopsy, *PTC* papillary thyroid cancer

^a^ *t* test was used for statistical analysis

All the patients qualified for surgical treatment were subjected to thyroid ultrasonography, determinations of free thyroid hormones (fT4, fT3), thyrotropic hormone (TSH), as well as anti-thyroid peroxidase (TPO) and anti-thyroglobulin (TG) antibodies. The inclusion criteria for surgery in the HT group included nodular lesions of the thyroid detected by ultrasound as hypoechoic or hyperechoic nodular pattern at least 5 mm in diameter, identification of a perinodular hypoechogenic or hyperechogenic halo, and the presence of an anechoic lesion with a reinforced posterior wall. A repeated analysis of fine-needle aspiration biopsy (FNAB) results based on the Bethesda 2009 classification showed that in the test results, the predominating lesions were these that today would be classified as groups III and IV of the above classification system. In the non-HT group, surgical treatment was indicated in case of suspicious lesions detected by FNAB (groups III–VI according to Bethesda 2009), symptoms of tracheal and surrounding tissue compression, multinodular toxic goiter, and Graves’ disease. Prior to surgical treatment, all the patients presenting with clinical and biochemical hypothyreosis received levothyroxine substitution. The mean follow-up time in the analyzed group was 4.3 ± 2.1 years. In the first postoperative day, indirect laryngoscopy was performed in all the patients and their calcium levels were determined; hypocalcemia was assumed at total calcium levels below 2.0 mmol/l. Following thyroidectomy and the diagnosis of differentiated thyroid carcinoma, a uniform model of therapeutic management was employed in all the patients; the model consisted of radioiodine adjuvant treatment in case of tumors with a diameter of >10 mm, administration of l-thyroxine (at a dose ensuring the suppression level for TSH range of 0.1–0.3 mU/l), and strict periodic follow-up determination of hormones and anti-TG.

Statistical analysis for normally distributed variables employed the Student’s *t* test in comparisons, while the remaining variables were analyzed using the *χ* ^2^ test. When the test assumptions were not fulfilled, comparisons were based on the so-called Fisher’s exact test. The criterion of including a variable into the model was *p* < 0.05.

## Results

In the analyzed group of 452 patients with Hashimoto thyroiditis, 106 (23.5 %) cases of papillary thyroid cancer (HT-PTC) were detected as compared to 530 (7.5 %) cancer patients in the group of 7,093 individuals (non-HT-PTC) without diagnosed autoimmune disease. The values are statistically significant (*p* < 0.001). Both the analyzed groups showed a similar ratio of males to females (1:9 in HT-PTC group vs. 1:10 in non-HT-PTC group) and a similar mean age of the patients (53.5 ± 12.1 years in HT-PTC group vs. 52.3 ± 15.5 years in non-HT-PTC group). A statistically significant difference was observed in preoperative goiter volume as determined by ultrasonography (22.1 ± 18.2 ml in HT-PTC group vs. 96.2 ± 40.4 ml in non-HT-PTC group, *p* < 0.001). FNAB was performed in all the patients with nodular lesions above 5 mm in diameter, but in the HT-PTC group, only 31 (29.2 %) of 106 HT-PTC subjects had thyroid tumor diagnosed preoperatively. In the remaining cases, cytological material did not raise any suspicions of neoplastic process. In FNAB smears, oxyphilic cells with lymphocytes and macrophages predominated (Table 1). In eight (12.3 %) patients (pT_1a_ = 65) with a nodule of 8–10 mm in size, FNAB confirmed thyroid microcarcinoma. In one case, in a 4-mm nodule, a focus of papillary thyroid carcinoma was detected preoperatively. The above lesions were seen in females in a lower age range as compared to the entire group (16–46 years).

However, prevalence of suspicious FNAB cytology in non-HT-PTC group was significantly higher and equal to 212 (40.0 %) of 530 patients (*p* < 0.001). pT_1a_ stage papillary thyroid carcinoma predominated in patients with Hashimoto thyroiditis as compared to the remaining analyzed patients. The values were 65 (14.4 %) in HT-PTC vs. 301 (4.24 %) in non-HT-PTC (*p* < 0.001). A similar distribution was observed in the group with the multifocal form of papillary thyroid cancer (pT_1m_): 22 (4.9 %) in HT-PTC vs. 119 (1.7 %) in non-HT-PTC, although the differences were not significant. Multifocality was defined as the presence of ≥2 cancer foci in one or both thyroid lobes. A statistically significant difference was noted with respect to nodal metastases in the central cervical compartment (compartment VI). Such metastases were observed in 81 of 106 (76.4 %) patients with HT-PTC and only in 121 of 530 (22.8 %) non-HT-PTC patients (*p* < 0.001). The metastases were particularly common in pT_1a_ papillary thyroid cancer: 46 (43.4 %) in HT-PTC vs. 29 (5.47 %) in non-HT-PTC. The mean size of the largest single focus in the HT-PTC group was 7.61 ± 5.78; nevertheless, in a high number of patients (pT_1m_: HT-PTC = 22 (4.9 %)), the lesions were 2–3 mm in size and were noted in both thyroid lobes (Table 2). In HT patients, preparations of cervical compartment lymph nodes showed a higher number of dissected lymph nodes and a higher number of metastatically involved lymph nodes as compared to non-HT patients (Table 2).

**Table 2:** Final histopathology diagnosis after thyroidectomy in 7,545 patients involved in this study

	HT (*n* = 452)	Non-HT (*n* = 7,093)	*p* value
Papillary thyroid cancer, no. (%)			
Total	106 (23.5)	530 (7.5)	<0.001
TNM classification			
Primary tumor			
pT_1a_N_x_M_x_	65 (61.3)	301 (56.7)	<0.001
pT_1b_N_x_M_x_	18 (17.0)	45 (8.4)	<0.001
pT_1m_N_x_M_x_	22 (20.8)	119 (22.4)	0.01
pT_2_N_x_M_x_	1 (0.9)	66 (12.5)	<0.001
Regional lymph nodes			
pT_1a_N_1a_M_x_	46 (43.4)	29 (5.5)	<0.001
pT_1b_N_1a_M_x_	16 (15.1)	41 (7.7)	<0.001
pT_1m_N_1a_M_x_	18 (17.0)	43 (8.1)	<0.001
pT_2_N_1a_M_x_	1 (0.9)	8 (1.5)	0.07
Diameter of the largest foci in mm (mean ± SD)	7.6 ± 5.8	9.4 ± 6.9	0.01
Number of removed lymph nodes within level VI (mean ± SD)	12.7 ± 3.7	6.5 ± 2.4	<0.001
Number of macrometastatic lymph nodes (mean ± SD)	6.4 ± 2.7	2.1 ± 1.0	<0.001

No significant differences were observed between HT-PTC patients and non-HT-PTC patients (Table 3) with regard to the prevalence of complications following thyroidectomy.

**Table 3:** Complications after thyroidectomy in 636 patients involved in this study

	PTC in HT (*n* = 106)	PTC in non-HT (*n* = 530)	*p* value
Parathyroid found in pathological report, no. (%)	5 (4.7)	27 (5.1)	0.871
Hypoparathyroidism after operation, no. (%)			
Total	47 (44.3)	209 (39.4)	0.347
Transient	45 (42.4)	197 (37.2)	0.306
Permanent	2 (1.9)	12 (2.3)	0.809
Unilateral RLN injury, no. (%)			
Total	12 (5.7)	55 (5.2)	0.766
Transient	9 (4.2)	41 (3.9)	0.792
Permanent	3 (1.4)	14 (1.3)	0.909

Calculation was made for nerves at risk, not for patients (there were 212 nerves at risk in PTC in HT group and 1,060 nerves at risk in PTC in non-HT group). Chi-squared test was employed for all values

## Discussion

An unambiguous association between papillary thyroid cancer and Hashimoto thyroiditis was demonstrated for the first time by Dailey et al. in 1955 [1]. According to abundant data from the literature on the subject [2–9, 17, 21–24, 34, 37, 38] and a meta-analysis performed by Singh et al. [2], PTC coexisted with Hashimoto thyroiditis 2.8 more frequently and its prevalence investigated in patient groups as reported in various articles ranged from 0.5 to 30 % (Table 4). PTC was also observed to occur almost twice as often as other types of thyroid cancer. In the presently analyzed material, in as many as 23.5 % patients, papillary thyroid cancer arose from lesions characterized as chronic autoimmune thyroiditis. This percentage constituted a threefold increase of the number of PTC cases in the population of Hashimoto thyroiditis patients as compared to the overall number of individuals with papillary thyroid cancer in the investigated group (non-HT-PTC = 530 patients). The above quoted data from literature and the results of our observations clearly point to an increase in neoplastic disease prevalence in patients suffering from an autoimmune disease. To date, there are no unambiguous indications for determining whether an autoimmune disease predisposes the patient to develop cancer or whether in the course of carcinogenesis-associated cellular transformations, there occurs a change in autoimmune responses that would facilitate the development of such a disease. Segal et al. [10] suggest that Hashimoto thyroiditis does not determine but rather delays cancer development, while circulating antibodies may constitute a significant factor preventing tumor development and nodal metastases. This thesis is in opposition to the investigations by Di Pasquale et al. [11] that suggest the very autoimmune background in all cases of cancer coexisting with Hashimoto thyroiditis. The hypothesis assumes an increased genetic predisposition to the development of neoplastic lesions. Information on molecular factors that determine initiation, promotion, and progression of thyroid cancers has been the subject matter of numerous investigations in recent years. In keeping with the theory of multistage carcinogenesis, various phenotypic variants of thyroid tumors may develop through genetic defects of the signaling cascade, via activation and inactivation of oncogenes and serial mutations. Presently, the best known form of such alterations in papillary thyroid cancer is the concept of oncogenic RET/PTC1 and RET/PTC3 sequences that are also present in chronic lymphocytic thyroiditis, but with no clinical manifestation of lesions in the thyroid parenchyma [12, 13]. The RET/PTC1 mutation was detected by Sheils et al. in as many as 95 % of investigated patients with Hashimoto thyroiditis [14]. In the aforementioned reports, the authors suggest that the presence of mutations involving the RET/PTC oncogenes may be an early molecular indicator of papillary thyroid microcarcinoma lesions. Recent experience with the use of PCR in cytological diagnostic management of material obtained by FNAB may be of importance in detecting neoplastic lesions before they become clinically manifested [15]. The significance of molecular studies as an element of cytological diagnostics of thyroid lesions is confirmed by considerable differences in papillary thyroid cancer diagnoses in biopsy materials. According to literature [2, 16–21], histology-confirmed diagnoses of papillary thyroid cancer fall within an extremely wide range from 0.4 % in the reports by Matesa et al. [17] to 92 % in keeping with the data provided by Singh et al. [2, 21]. In our material, in 31 of 452 (6.9 %) HT patients, confirmation of the diagnosis of papillary thyroid carcinoma in material collected by targeted fine-needle aspiration biopsy was achieved, which accounted only for 29.2 % of all HT-PTC diagnoses established based on final histopathology. The reason behind such significant differences lies in difficulties in defining lesions that require cytological diagnostics. In all nodular, localized lesions situated within the thyroid parenchyma, specificity and sensitivity were high, ranking between 87 and 92 %. In atrophic Hashimoto thyroiditis with increased tissue density, false-negative results predominated. This happened due to lymphocytic infiltrations with reaction center formation both in the thyroid tissue and around the suspected primary focus. Some authors believe the presence of such infiltrations to be a factor predisposing to tumor development [2, 22], while others regard it as a response of the body to neoplastic disease, hence the potentially better prognosis in this group of patients [2, 9, 23–25]. Kebebew et al. [23] firmly indicate that the presence of chronic lymphocytic thyroiditis in patients with papillary thyroid cancer improves the prognosis. On the other hand, it cannot be at the same time an independent prognostic factor indicating a lower number of recurrences or the presence of nodal metastases. This common transformation path of thyroid cells in papillary thyroid cancer and Hashimoto thyroiditis has been also attempted to be explained by similarities in activation of the metabolic cycle of tyrosine kinases (PI3k/Akt pathway) and overexpression of p63 protein that leads to apoptosis inhibition [26, 27].

**Table 4:** Coexistence Hashimoto thyroiditis with papillary thyroid cancer

Authors	Patient included in the study	HT with PTC, *n* (%)
Dailey at al. [1]	278 with PTC	35 (12.6)
Singh et al. [2]	388 with PTC	57 (15.0)
Chesky VE et al. [3]	432 with HT	48 (11.1)
Ott et al. [6]	161 with TC	61 (38.0)
Eisenberg et al. [7]	120 with TC	13 (10.8)
Sclafani et al. [8]	48 with HT	8 (17.0)
Schäffler et al. [9]	153 with TC	10 (6.5)
Matesa-Anić et al. [17]	10,508 FNAB	42 (0.5)
Cipolla et al. [20]	71 with PTC	19 (26.7)
Pisanu et al. [21]	344 with PTC	33 (9.6)
Kebebew et al. [23]	136 with PTC	41 (30)
Matsubayashi et al. [24]	95 with PTC	36 (37.9)
Yoon et al. [34]	195 with PTC	56 (28.7)
Paulson et al. [37]	139 with PTC	61 (43.8)
Repplinger et al. [38]	217 with HT	63 (29)

Literature review was used in this study

*HT* Hashimoto thyroiditis, *TC* thyroid cancer, *PTC* papillary thyroid cancer, *FNAB* fine-needle aspiration biopsy

Another extremely important element that plays a role in clinical assessment of papillary thyroid cancer is the size of the primary focus. Tumors up to 10 mm in diameter are characterized by a favorable prognosis, although they may demonstrate all specific properties of a malignancy, with possible nodal metastasizing, capsular infiltration, or multifocal growth in the thyroid tissue. Bradley et al. [28] analyzed a group of accidentally detected forms of papillary thyroid cancer (687 patients, 81 IPTC (12 %)) and confirmed this tumor form in as many as 21 (28 %) patients of 74 individuals with Hashimoto thyroiditis. The primary focus size did not exceed 7 mm. (mean 2.8 ± 1.7 mm). In the present analysis, although the mean focus size was larger as compared to the above-mentioned values, it did not exceed 10 mm in diameter, thus providing the basis for microcarcinoma diagnosis. In the analyzed material, microcarcinomas were markedly predominant (65 cases = 61.3 %). Of significance is also a relatively high number (22 = 20.8 %) of patients with multifocal PTC form and with foci with the diameter starting from 2 to 5 mm. Based on the analysis, one may thus surmise that coexistence of an autoimmune disease and papillary thyroid cancer did not limit the number of primary foci and affected their size only [29, 30]. The above data have been quite extensively confirmed in the literature on the subject. Numerous authors are inclined to believe that coexistence of lymphocytic infiltrations, positive history of thyroid autoimmune diseases, or significantly high antibody levels (anti-TPO, anti-TG) prior to surgery had a significant impact on prognosis improvement. Although there have been voiced opinions that such factors did not affect mortality rates, they undoubtedly determined early initiation of targeted therapy [30].

Tumor grading determined by the degree of capsular infiltration and nodal metastasizing allows for selecting patients with a poor prognosis. As it has been mentioned previously, papillary thyroid cancer is generally characterized by a good prognosis. This is particularly true in patients with highly differentiated pT_1a_ cancers that do not exceed 10 mm in diameter. It should be noted, however, that even these forms show a fairly high ability to metastasize. In their analysis of 203 patients with papillary thyroid microcarcinoma, So et al. found nodal metastases in as many as 24.6 % of subjects, multifocal cancer in 31 %, and capsular infiltrations in 20.7 % of their surgical patients [31]. Similar observations were made by Mercante et al. and Cho et al. [32, 33], who emphasized that the size of the primary focus did not play a role in the presence or absence of nodal metastases or capsular infiltrations. Is there, then, any difference in tumor morphology, ability to metastasize, and prognosis in patients with papillary thyroid cancer and Hashimoto thyroiditis?

In the entire presently analyzed material, the presence of central compartment lymph node metastases was significantly higher in patients with cancer and Hashimoto thyroiditis (PTC-HT = 81 (76.4 %) vs. non-HT-PTC = 121 (22.8 %)). According to numerous reports, metastatic foci in cervical lymph nodes accompany both “clear” cancer forms and tumors presenting with symptoms of chronic lymphocytic thyroiditis. The degree of nodal involvement—differently reported in various publications—ranged from 7 to 49 % [34–36]. In one of the recent articles, Paulson et al. [37] suggest that a lower number of nodal metastases to the central compartment are a result of a coexisting autoimmune disease and the protective role it plays in neoplastic spreads beyond the primary focus area.

Based on our observations of both groups of patients, a conclusion has been formulated that concomitant Hashimoto’s thyroiditis and papillary thyroid carcinoma are more common phenomena than previously believed, since they are observed in approximately 20 % of all cases of this cancer type. According to data from the literature on the subject, it is significantly more common among women from younger age groups, is characterized by lesions with a small diameter of the primary focus, and, at times, has a multifocal form, but the prognosis is statistically more favorable as compared to other patient groups [20, 38]. Improved ultrasound techniques, higher availability of examinations, and higher awareness of patients today allow for early institution of appropriate treatment. The above-described observations of the present authors, as well as data from world literature, indicate a need for further immunological and genetic investigations aiming at understanding the nature of a protective role of coexistence of an autoimmune disease and thyroid cancer. It should be remembered, however, that the continuously low number of patients, high costs of genetic probes, and ambiguous diagnostic protocol limit the use of such observations in clinical practice. Hence, in view of the high percentage of PTC in HT and incidence of complications following total thyroidectomies that is comparable to that observed in operations performed in patients with nonneoplastic goiter [39–41], the optimum strategy of surgical treatment of Hashimoto thyroiditis in such patients should include total thyroidectomy and preventive central compartment lymphadenectomy, which is of importance in assessing tumor stage and determining recommendations for further adjuvant therapy. Total thyroidectomy along with excision of central compartment lymph nodes may result in damaging recurrent laryngeal nerves and accidental excision of inferior parathyroids glands. Nevertheless, the incidence of such complications in referral centers is low.

The literature on the subject increasingly more often presents reports stating that primary total thyroidectomy allows not only for treating the neoplastic disease already diagnosed based on the FNAB result but also contributes to a decrease in reoperation rate due to postoperative diagnosis of thyroid cancer. Analyzing this aspect, we may say that primary total thyroidectomy also limits the number of permanent complications, which in a significant percentage of cases pose a considerable threat in the course of reoperation. In our opinion, the above-described rationale provides a foundation for considering radical surgical treatment in cases of nodular lesions being present in immunologically altered thyroid gland.
